# STAT1 and its related molecules as potential biomarkers in *Mycobacterium tuberculosis* infection

**DOI:** 10.1111/jcmm.14856

**Published:** 2020-02-12

**Authors:** Xing‐hao Yi, Bo Zhang, Yu‐rong Fu, Zheng‐jun Yi

**Affiliations:** ^1^ Key Laboratory of Clinical Laboratory Diagnostics in Universities of Shandong Department of Laboratory Medicine and clinical medical collegue Weifang Medical University Weifang China; ^2^ Clinical Medical College Jining Medical University Jining China; ^3^ Weifang No. 2 People's Hospital Weifang China

**Keywords:** bioinformatic analysis, biomarkers, circRNA, miRNA, tuberculosis

## Abstract

Tuberculosis (TB) is a severe infectious disease that seriously endangers human health. The immune defence mechanism of the body against TB is still unclear. The purpose of this study was to find the key molecules involved in the immune defence response during TB infection, and provide reference for the treatment of TB and further understanding of the immune defence mechanism of the body. Data from http://www.ncbi.nlm.nih.gov/geo/query/acc.cgi?acc=GSE83456 were downloaded from GEO data sets for analysis, and a total of 192 differentially expressed genes were screened out. Most of these genes are enriched in the interferon signalling pathway and are defence response–related. We also found that STAT1 plays an important role in the immune defence of TB infection and it is one of the key genes related to interferon signalling pathway. STAT1‐related molecules including hsa‐miR‐448, hsa‐miR‐223‐3p, SAMD8_hsa_circRNA 994 and TWF1_hsa_circRNA 9897 were therefore screened out. Furthermore, expression levels of hsa‐miR‐448 and hsa‐miR‐223‐3p were then verified by qRT‐PCR. Results showed that both hsa‐miR‐448 and hsa‐miR‐223‐3p were down‐regulated in plasma from patients with pulmonary TB. Taken together, our data indicate that an mRNA‐miRNA‐circRNA interaction chain may play an important role in the infection of MTB, and STAT1 and related molecules including hsa‐miR‐223‐3p, has‐miR‐448, SAMD8_hsa_circRNA994 and TWF1_hsa_circRNA9897 were identified as potential biomarkers in the development of active TB.

## 1 INTRODUCTION

Tuberculosis (TB) is a chronic infectious disease in which clinical manifestations appear as prolonged low fever, expectoration and haemoptysis.^1^ *Mycobacterium tuberculosis* (MTB) is the pathogenic bacterium of TB. Pulmonary TB is the most common type of TB infectious. In addition to lung tissue, MTB can also infect the enteric canal, lymph gland, articulatio, spinal, urogenital system and other organs or tissues, therefore caused dysfunction and pathological damage of human body.^2^

In recent years, multidrug‐resistant tuberculosis (MDR‐TB) and the co‐infection of TB and AIDS have made TB treatment approach even more rigorous. The WHO report on global tuberculosis shows that 1.6 million people (including 300 000 people with HIV) and 2.3 million children died from TB (including children with HIV‐related TB) in 2017. Meanwhile, of the 5.58 million new rifampicin‐resistant cases, 82% were MDR‐TB.^3^ The occurrence, development and outcome of TB are not only related to the toxicity and quantity of bacteria, but also closely related to the immune function of the body. MTB is an intracellular parasitic bacteria, and the body's anti‐TB immunity is mainly cellular immunity. The synergistic effect of cytokines with various immune cell subtypes including CD4^+^, CD8^+^ and NK cells plays a key role in the immune defence of tuberculosis, the most important of which are macrophages, effector CD4^+^ T lymphocytes and IFN‐γ, which is secreted by Th1 cells and induces macrophage activation.^4^

However, MTB can hinder oxidative stress, apoptosis and autophagy as well as inhibits the synthesis of histocompatibility complex molecules and thus affects antigen presentation. These mechanisms inhibit and resist the macrophages' natural immune killing and specific immune response, so as to help MTB escape from the body's immune killing.^5^ Therefore, a full understanding of the immune response mechanism of MTB infection has important theoretical significance for the clinical diagnosis as well as the research of new TB vaccine and immunotherapy.^6^ The immune response in the blood can reflect the local response of the lung to pathogens, so the changes in whole blood composition can be used as a sensitive indicator in TB infection.^7^ Study has shown that transcriptional signature of active TB reflects symptom status in pulmonary TB, the researchers have compared the transcriptional signature of healthy people, patients with pulmonary TB, patients with extrapulmonary TB and patients with sarcoidosis, and the meta‐signature shows that it can differentiate active TB from healthy controls but the results in distinguishing TB from extrapulmonary TB were less than ideal.^8^ However, in this study, we used bioinformatic methods to compare and analyse the original genetic data of the whole blood of the patients with pulmonary TB and healthy people, hope to investigate potential miRNAs and circRNAs that may play crucial roles in TB, so as to reveal the pathogenesis of TB at the molecular level and excavate potential biomarkers of TB.

## 2 MATERIALS AND METHODS

### 2.1 Acquisition of RNA information

The clinical samples of TB infection were retrieved from the GEO database. A total of 106 blood samples were selected from http://www.ncbi.nlm.nih.gov/geo/query/acc.cgi?acc=GSE83456, including 45 pulmonary tuberculosis (PTB) samples and 61 healthy control (HC) samples.^8^ Pulmonary TB patients met the operational diagnosis of TB infection. Patients who are suffering from immunosuppressed diseases such as HIV, diabetes or autoimmune diseases, medication or had comorbidities that affecting the pulmonary system were excluded. All RNA information of the selected samples was downloaded for further analysis. The sample's information and data used in this section were all downloaded from public database; therefore, no patient consent or ethics committee approval was necessary.

### 2.2 Data process

The original expression matrix was normalized and processed by R. The limma package was used to screen out differentially expressed genes.^9^ The *P*‐value of genes was calculated using *t* test method, and Benjamini and Hochberg's method was used to calculate the adjusted *P*‐value. The differentially expressed genes were screened out by the following selection criteria: at least a 2.0‐fold change between healthy controls and pulmonary TB patient samples and with adjusted *P*‐value < .05.

### 2.3 Enrichment analysis

Gene set enrichment analysis (GSEA) sequenced the genes according to the differential expression degree of the two samples and then detected whether the preset gene set was enriched at the top or bottom of the sequencing table.^10^ The analysis tests the expression of genomic rather than individual genes and can therefore include more subtle changes in expression. All genetic information of PTB and HC samples was uploaded to GSEA for further analysis. Database for annotation, visualization and integrated discovery (DAVID) v6.8 was used to analyse the differential expression genes in PTB, including molecular function (MF), biological process (BP) and cell composition (CC).^11^ In addition, differential gene pathway analysis was performed in Functional Enrichment analysis tool (Funrich).^12^ Pathway analysis was conducted to find out which cell pathways might be involved in the changes in differentially expressed genes. Therefore, crucial pathway related to differentially expressed genes can be identified. The pathway analysis was validated using IPA software.

Ingenuity Pathway Analysis (IPA) is a software which can show the activation level of biological pathways, and because the database is updated once a week, it has high credibility and accuracy, which is a great help for bioinformatic analysis.^13^ Therefore, we uploaded the 192 differentially expressed genes to IPA to perform canonical pathway and molecule function analysis. The statistical cut‐off of the enriched functions or pathways was determined by Fisher's exact test *P*‐value, which assesses whether correlations between meaningful molecules and known processes come from random matches, and the *z*‐score, which evaluate the directional effects of one molecule on another or the effects of multiple molecular changes in the data set on biological processes, thus assess the match of observed and predicted regulatory patterns with a prediction for the activation state. Both *P*‐value < .05 and absolute value of *z*‐score > 2 are considered significant.

### 2.4 Gene cluster identification and protein‐protein interaction (PPI) network analysis

The differential genes in PTB samples were uploaded to STRING to obtain the protein network interaction diagram.^14^ The result of STRING analysis was imported into Cytoscape v.3.7.1, and cluster analysis of differential genes was conducted using Molecular Complex Detection (MCODE) plug‐in.^15^ The genes contained in the gene cluster with the highest scores were imported into the STRING to draw the protein interaction network and further analyse which biological processes this gene cluster was participated in. The screened gene cluster was then uploaded to Network Analyst for further verification.^16^

### 2.5 Prediction of pivotal miRNAs and construction of gene‐miRNA interaction network analysis

Genes related to the crucial pathway were selected and performed with miRWalk 2.0 to predict its targeted miRNAs.^17^ To verify the accuracy of the results, five databases including TargetScan, miRanda, miRDB, miRWalk and RNA22 were used to do intersection. The final result obtained from the intersection is further processed with Cytoscape v 3.7.1; therefore, miRNAs which targeted more than two genes are selected.

### 2.6 Quantitative reverse transcription polymerase chain reaction (qRT‐PCR)

Two candidate plasma biomarkers for TB disease were screened for further confirmation. For this, a total of 18 participants, including 11 TB patients and 7 healthy volunteers, were recruited from Weifang No. 2 People's Hospital and Weifang Medical University, and there was no significant difference in age and gender between them (age: 20‐52). TB patients were diagnosed based on sputum smear or culture positive and clinical symptoms, who were excluded if he or she had the following diseases such as cancer, diabetes, HIV or HBV infection, or other lung diseases. Informed consent was obtained from all patients prior to beginning the study.

Plasma sample was collected from each participant before initial therapy. Subsequently, total RNA was extracted from per sample using TRIzol (Invitrogen), and then, its concentration and purity were assessed by K5800 Micro‐spectrophotometer (Kaiao). The reverse transcription was conducted using PrimeScript™ 1st Strand cDNA Synthesis Kit (Takara) at 42℃ for 60 minutes and then at 95℃ for 5 minutes. Next, based on LightCycler^®^ 480 II real‐time PCR system (Roche), PCR was performed with SYBR^®^ Premix Ex Taq™ Kit (Takara) at the temperature of 95℃ for 2 minutes, followed by 38 cycles with the temperature of 95℃ for 30 seconds, 53℃ for 30 seconds and 72℃ for 30 seconds. U6 was applied as internal controls. The 2^−ΔΔCt^ method was utilized to determine the relative expression of each selected miRNA between case and controls. Sequences of primers used in the study are shown in Table 1.

**Table 1:** Oligonucleotides used in this study

Primer sets name	Reverse transcriptase primer (5′ to 3′)	Real‐time quantitative PCR primer (5′ to 3′)
U6	GTCGTATCCAGTGCAGGGTCCGAGGTATTCGCACTGGATACGACAAAATA	F: AGAGAAGATTAGCATGGCCCCTG
		R: ATCCAGTGCAGGGTCCGAGG
hsa‐mir‐223‐3p	GTCGTATCCAGTGCAGGGTCCGAGGTATTCGCACTGGATACGACTGGGGT	F:GCGCGTGTCAGTTTGTCAAAT
		R:AGTGCAGGGTCCGAGGTATT
hsa‐mir‐448	GTCGTATCCAGTGCAGGGTCCGAGGTATTCGCACTGGATACGACATGGGA	F:GCGCGTTGCATATGTAGGATG
		R:AGTGCAGGGTCCGAGGTATT

### 2.7 miRNAs‐circRNA prediction

StarBase v2.0 tool was used to predict the upstream molecules circRNAs of the selected miRNAs, and the obtained data were processed using Cytoscape software.^18^ The intersection of predicted results of each miRNA was obtained by using the cross‐linked graph to identify relevant circRNAs.

## 3 RESULTS

### 3.1 Sample information processing and screening of differentially expressed genes

**Figure 1:**
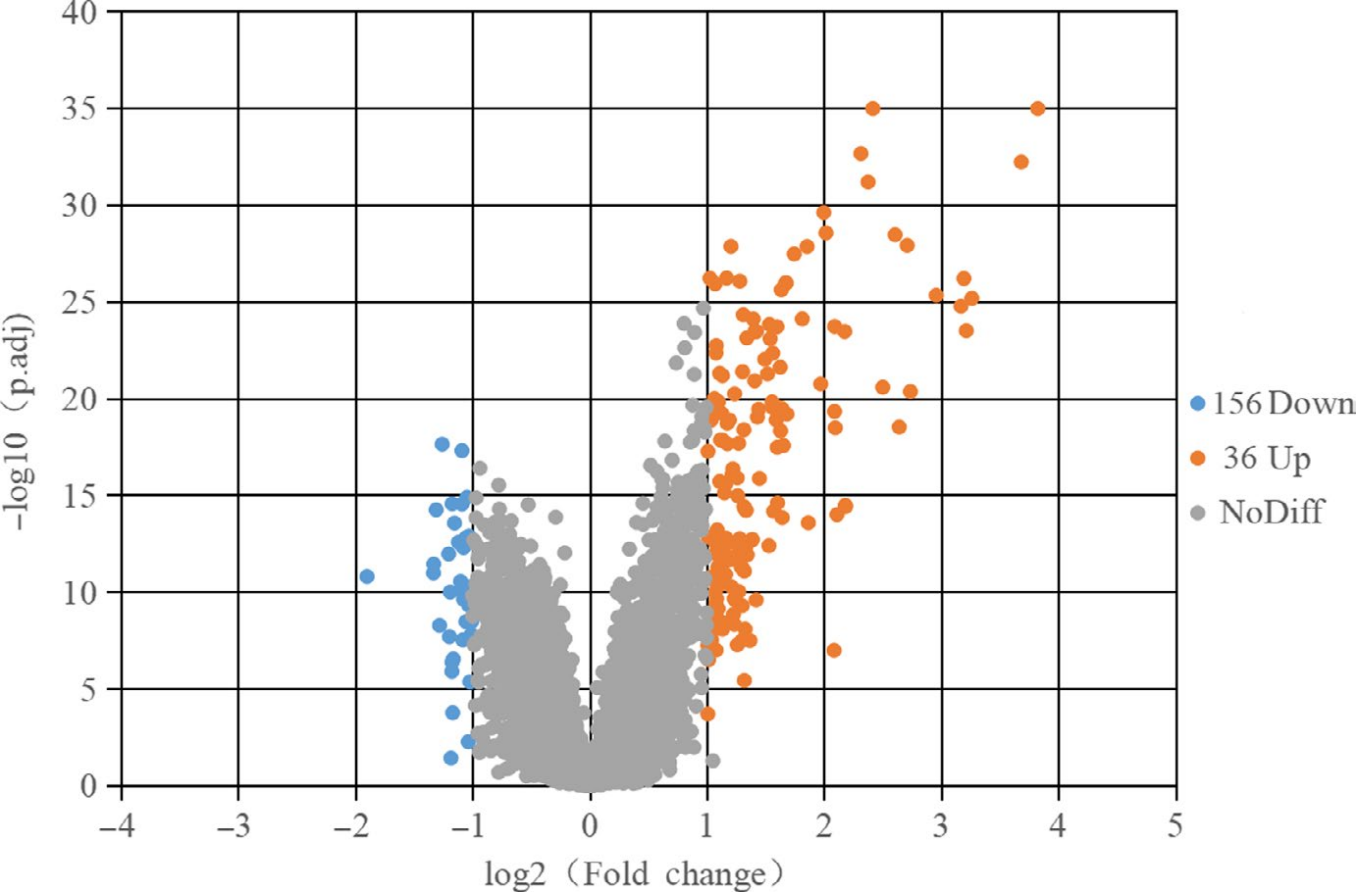
Differentially expressed genes in PTB and HC samples were shown in the volcano plot, with blue dots representing significantly down‐regulated genes in PTB samples and orange dots representing significantly up‐regulated genes. HC, healthy control; PT, pulmonary tuberculosis

According to the sample information and data matrix, 192 differentially expressed genes were extracted from the PTB samples, among which 156 genes were up‐regulated and 36 genes were down‐regulated. The screening criteria for differentially expressed genes were as follows: adjust *P*‐value < .05 and log _2_ fold change > 1. Based on the analysis of gene expression of the samples, the volcano plot was made as shown in Figure 1.

### 3.2 The differentially expressed genes in the PTB samples were mainly enriched in interferon (INF) signalling pathway and immune response

**Figure 2:**
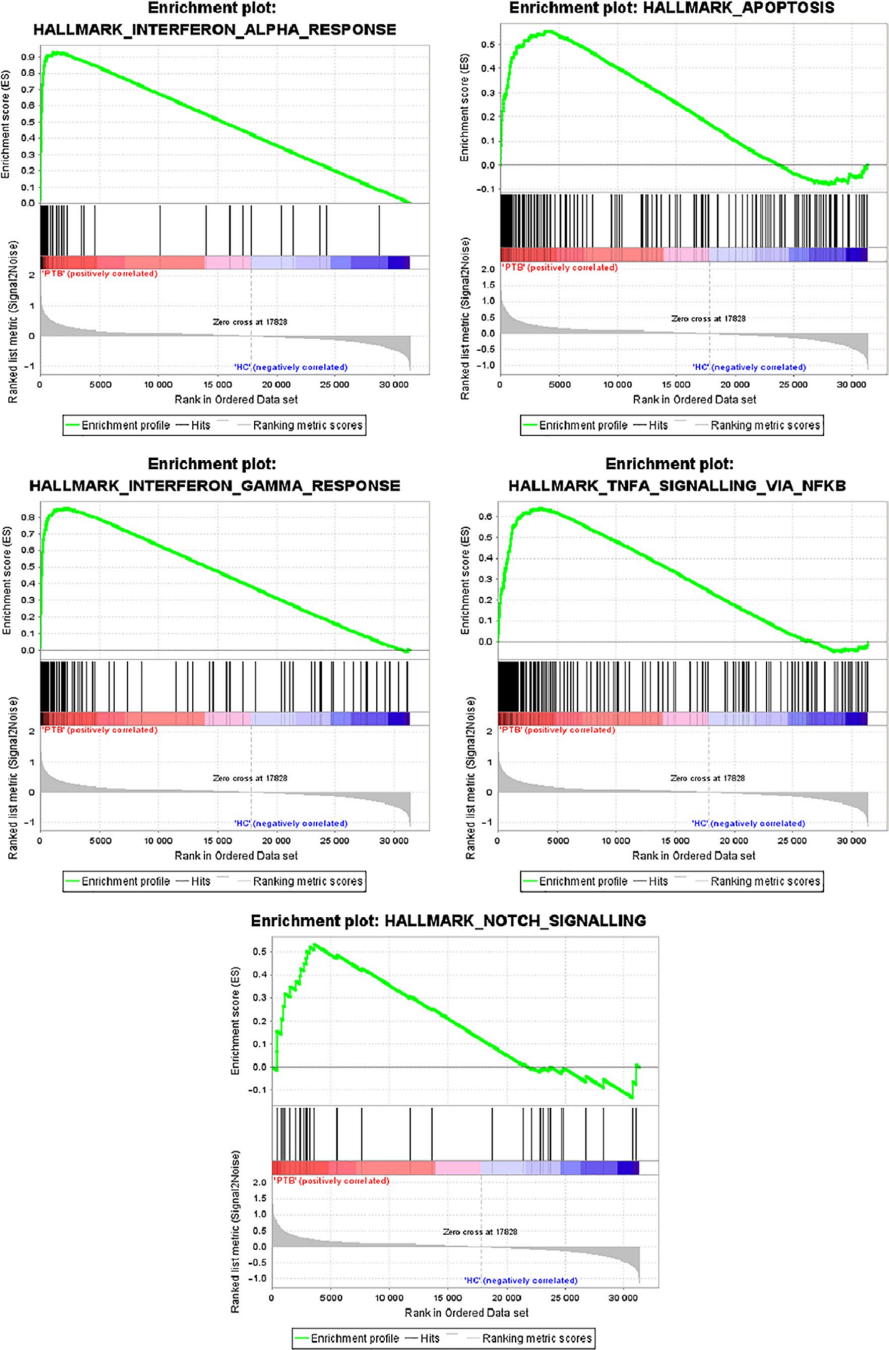
h.all.v 6.2.symbols.gmt [Hallmarks] gene set database was used to analyse the whole gene expression value of the PTB and HC samples. GSEA first filtered the gene set according to the number of genes contained in the gene set, with the minimum number of 15 genes and the maximum number of 500 genes by default. Significant gene sets were cut‐off by FDR < 0.25 and *P*‐value < .05

Gene set enrichment analysis, DAVID and Fun Rich software were used for enrichment analysis of the samples’ genes. Firstly, all gene expression information in PTB and HC samples was uploaded to GSEA software, and the hallmark gene set database was used to analyse genes at the overall level of expression profile. The significantly enriched gene sets were set at a default cut‐off as *P*‐value < .05 and FDR < 0.25. The enrichment analysis of gene sets revealed that the gene sets were significantly enriched in interferon‐alpha/gamma response, and immune‐related functions were significantly enriched in PTB samples as shown in Figure 2.

**Figure 3:**
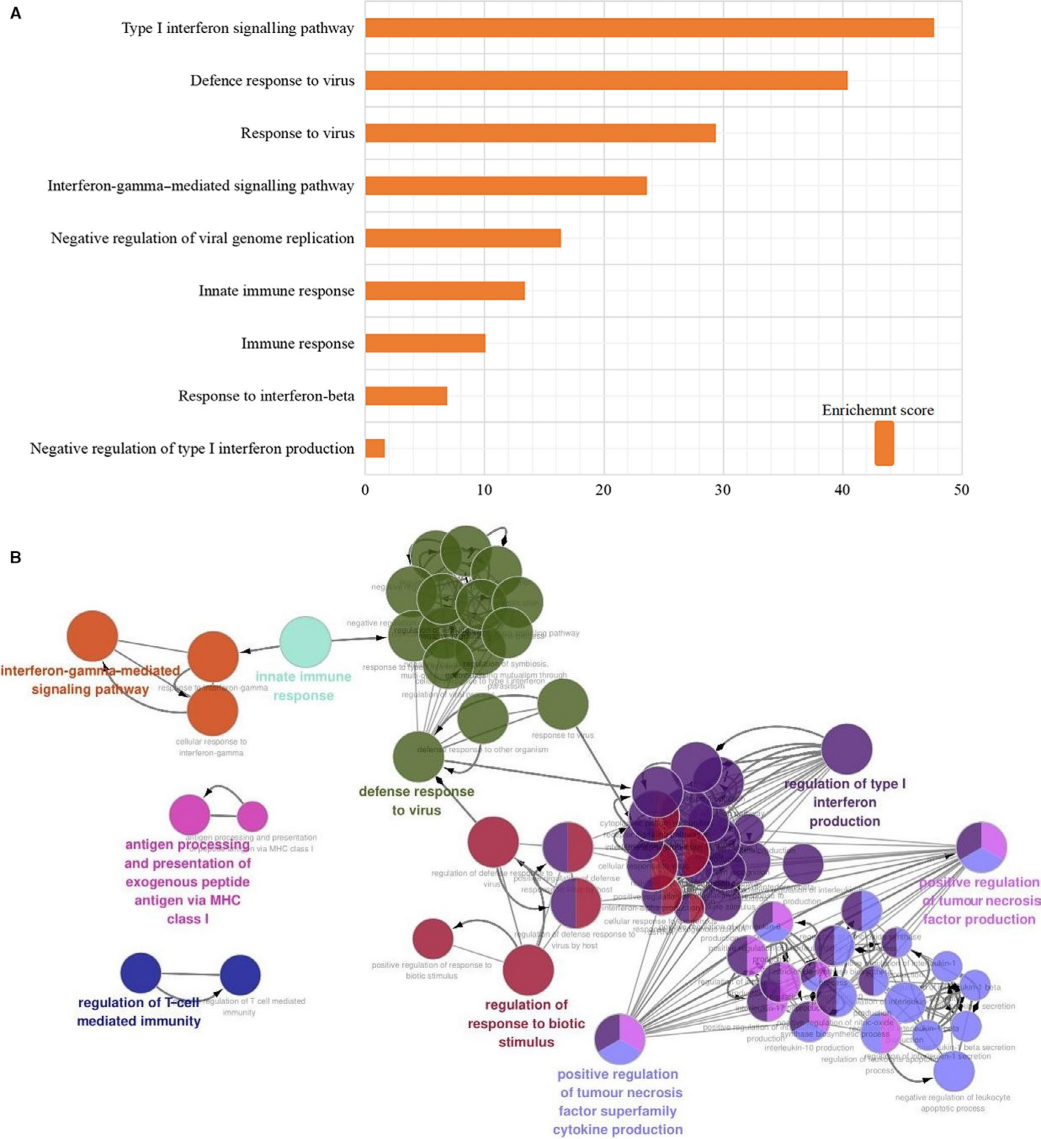
A, Top 9 biological processes were selected and shown in bar chart according to enrichment score. B, Use ClueGO to analyse the interaction networks of enriched biological processes, and multiple colour dots indicate that it revolved in multiple biological processes

GO and pathway enrichment analysis was conducted on 192 differentially expressed genes in the PTB samples by using DAVID and Funrich software. GO enrichment analysis showed that the differentially expressed genes in PTB samples were mainly related to interferon signalling and immune response in the biological process. The top nine biological processes were screened out according to the *P‐*value < .05, and the bar chart was drawn according to the enrichment score. The results shown in Figure 3A indicated that the significantly enriched biological process was type I interferon signalling pathway. Use Cytoscape ClueGo plug‐in to visualize the interaction network of biological process, as shown in Figure 3B.

**Figure 4:**
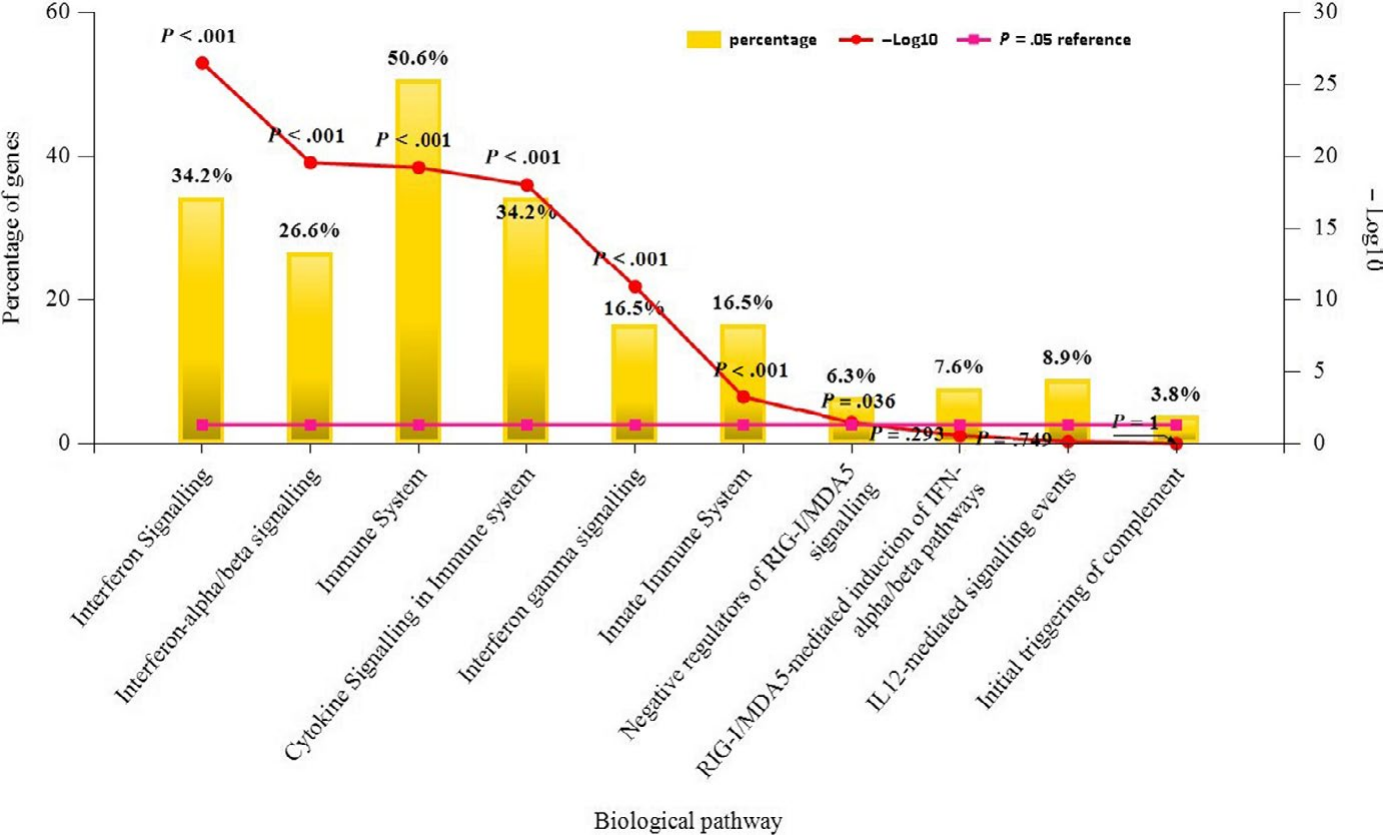
The Funrich software drew a bar chart of 10 biological pathways based on the *P*‐value and the percentage of genes, among which biological pathways with *P*‐value < .05 are statistically significant. The results showed that the biological pathways with significantly enriched were immune system–related

A total of 192 differentially expressed genes were then uploaded to Funrich, among which 163 genes were identified for further enrichment analysis. Through Funrich analysis of the pathway enrichment of differentially expressed genes in PTB samples, it indicated that the differentially expressed genes are mainly enriched in the interferon signalling pathway and immune‐related pathways as shown in Figure 4.

**Figure 5:**
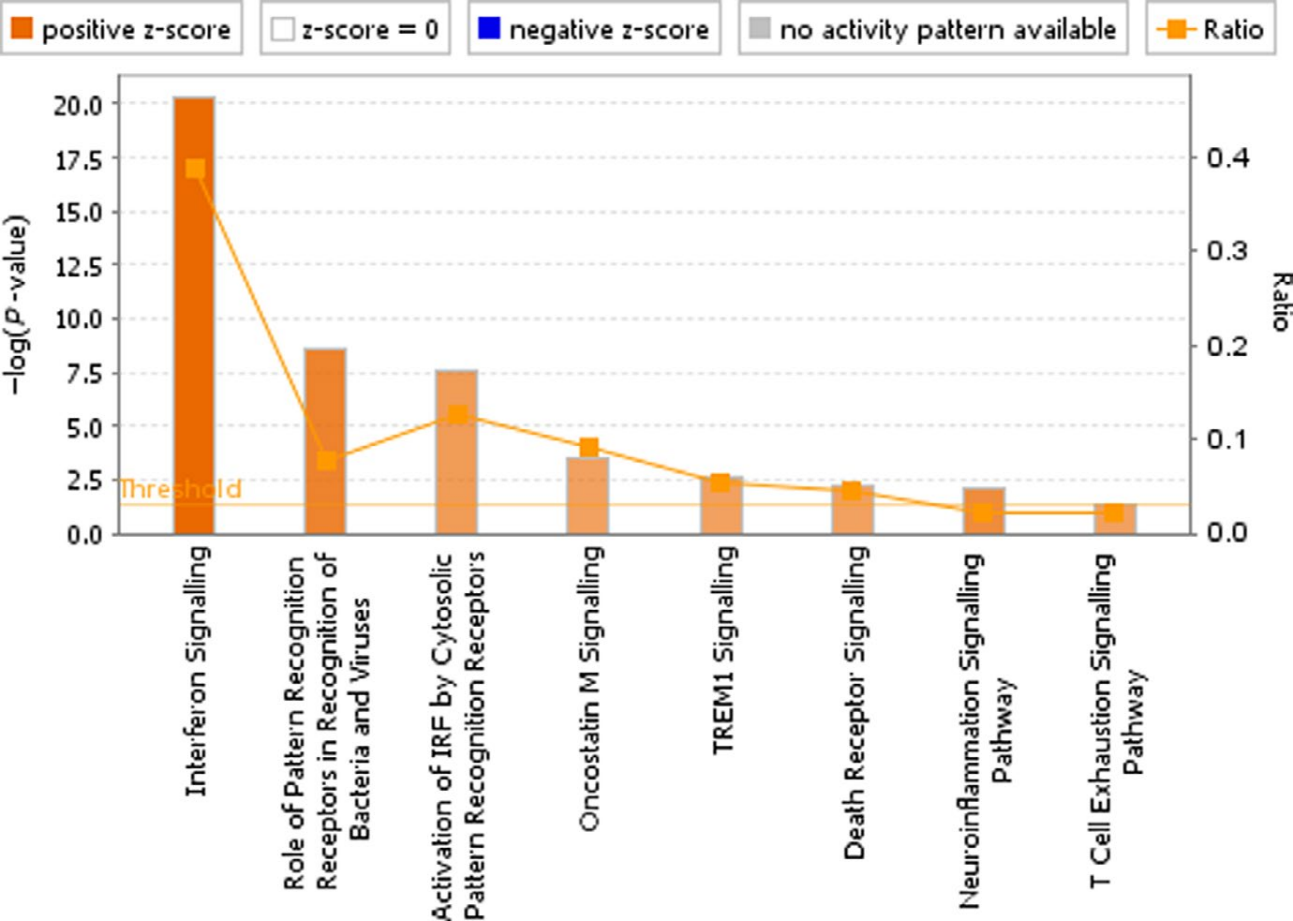
The canonical pathway analysis of IPA. Results show that there are total of 8 pathways were highly activated, especially the interferon signalling pathway which had the highest activation scores (*P*‐value = 4.27E‐21, *z*‐score = 3.207). The remaining high activation pathways include the role of pattern recognition receptors in recognition of bacteria and viruses (*P*‐value = 2.64E‐09, *z*‐score = 2.646), activation of IRF by cytosolic pattern recognition receptors (*P*‐value = 6.3E‐08, *z*‐score = 2.121), oncostatin M signalling (*P*‐value = 3.19E‐04, *z*‐score = 2.000), TREM1 signalling (*P*‐value = 2.6E‐03, *z*‐score = 2.000), death receptor signalling (*P*‐value = 5.21E‐03, *z*‐score = 2.000), neuroinflammation signalling pathway (*P*‐value = 8.48E‐03, *z*‐score = 2.449) and T cell exhaustion signalling pathway (*P*‐value = 4.61E‐02, *z*‐score = 2.000). The depth of the colours in the bar chart is based on the *z*‐score, and generally, an absolute *z*‐score greater than 2 is considered meaningful

**Figure 6:**
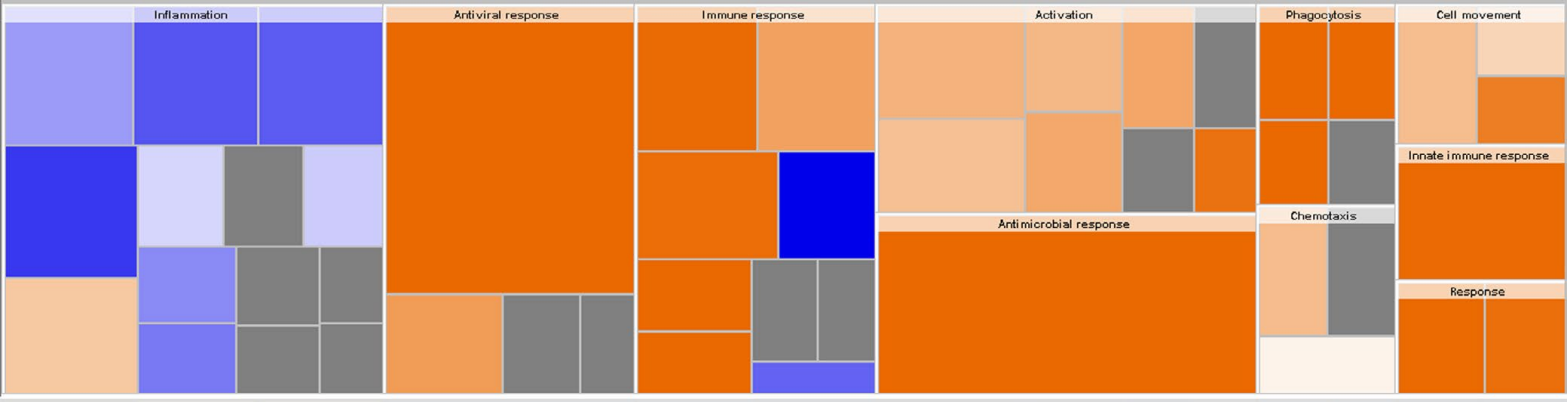
The biological function analysis of IPA. Results show that there are total of 10 main functional modules were associated with the differentially expressed genes. The names of these 10 modules are inflammation, antiviral response, immune response, activation, antimicrobial response, phagocytosis, chemotaxis, cell movement, innate immune response and response. Among all the subdivision function modules of these 10 main function modules, the relevant functions that are significantly activated are phagocytosis of cells (*P*‐value = 1.05E‐03, *z*‐score = 2.941), immune response of macrophages (*P*‐value = 5.59E‐05, *z*‐score = 2.621), antiviral response (*P*‐value = 2.96E‐35, *z*‐score = 2.411) and innate immune response (*P*‐value = 2.40E‐11, *z*‐score = 2.157). The obvious inhibitory functions are immune response of brain (*P*‐value = 5.06E‐05, *z*‐score = −2.399) and encephalitis (*P*‐value = 1.89E‐04, *z*‐score = −2.212). The biological function analysis shows the enrichment of differential genes in biological function classification, ranking from high to low according to the ‐log (*P*‐value) value (ie ranking from small to large according to the *P*‐value), and the heat map of biological function shows that the up‐regulated expression of differential genes is related to the activation or inhibition of biological function. Orange means *z*‐score > 0, blue means *z*‐score < 0, and grey means no *z*‐score; *Z*‐score > 2 means that the function is significantly activated, and *z*‐score < −2 means that the function is significantly inhibited

To further validate our results and to identify crucial molecules that involved in the progress of TB infection, a total of 192 differentially expressed genes were uploaded to IPA for core analysis. The canonical pathway results show that a total of 8 pathways including the interferon signalling pathway (*P*‐value = 4.27E‐21, *z*‐score = 3.207), role of pattern recognition receptors in recognition of bacteria and viruses (*P*‐value = 2.64E‐09, *z*‐score = 2.646), activation of IRF by cytosolic pattern recognition receptors (*P*‐value = 6.3E‐08, *z*‐score = 2.121), oncostatin M signalling (*P*‐value = 3.19E‐04, *z*‐score = 2.000), TREM1 signalling (*P*‐value = 2.6E‐03, *z*‐score = 2.000), death receptor signalling (*P*‐value = 5.21E‐03, *z*‐score = 2.000), neuroinflammation signalling pathway(*P*‐value = 8.48E‐03, *z*‐score = 2.449) and T cell exhaustion signalling pathway (*P*‐value = 4.61E‐02, *z*‐score = 2.000) are highly activated (Figure 5). Among these pathways, the interferon signalling pathway had the highest activation scores (*z*‐score = 3.07, *P*‐value = 4.27E‐21), and a total of 14 genes are related to this pathway including STAT1, MX1, OAS1, SOCS1, STAT2, TAP1, IFI6, IFI35, IFIT1, IFIT3, IFITM1, IFITM3, ISG15 and JAK2. Biological function analysis shows that the differentially expressed genes are associated with total of 10 main functional modules, including inflammation, antiviral response, immune response, activation, antimicrobial response, phagocytosis, chemotaxis, cell movement, innate immune response and response (Figure 6). The IPA shows that among these ten main functional modules, the subdivision function modules that are highly activated are immune response of phagocytosis of cells (*P*‐value = 1.05E‐03, *z*‐score = 2.941), immune response of macrophages (*P*‐value = 5.59E‐05, *z*‐score = 2.621), antiviral response (*P*‐value = 2.96E‐35, *z*‐score = 2.411) and innate immune response (*P*‐value = 2.40E‐11, *z*‐score = 2.157). The obvious inhibitory functions are immune response of brain (*P‐*value = 5.06E‐05, *z*‐score=−2.399) and encephalitis (*P*‐value‐1.89E‐04, *z*‐score=−2.212). Upstream analysis shows that the top five upstream regulators are STAT1 (*P*‐value = 9.81E‐61, *z*‐score = 6.392), IRF7 (*P*‐value = 2.77E‐57, *z*‐score = 6.532), IFNL1 (*P*‐value = 1.22E‐56, *z*‐score = 5.725), IFNG (*P*‐value = 3.33E‐52, *z*‐score = 7.742) and IFNA2 (*P*‐value = 1.99E‐48, *z*‐score = 6.241), among which, the first two are transcription regulators and the last three are cytokines. In addition, interferon alpha also shows a high activation *z*‐score of 5.839 and a *P*‐value of 7.80E‐47.

### 3.3 Construction of protein‐protein interaction (PPI) network and further excavation of gene clusters involved in immune system–related biological pathway

**Figure 7:**
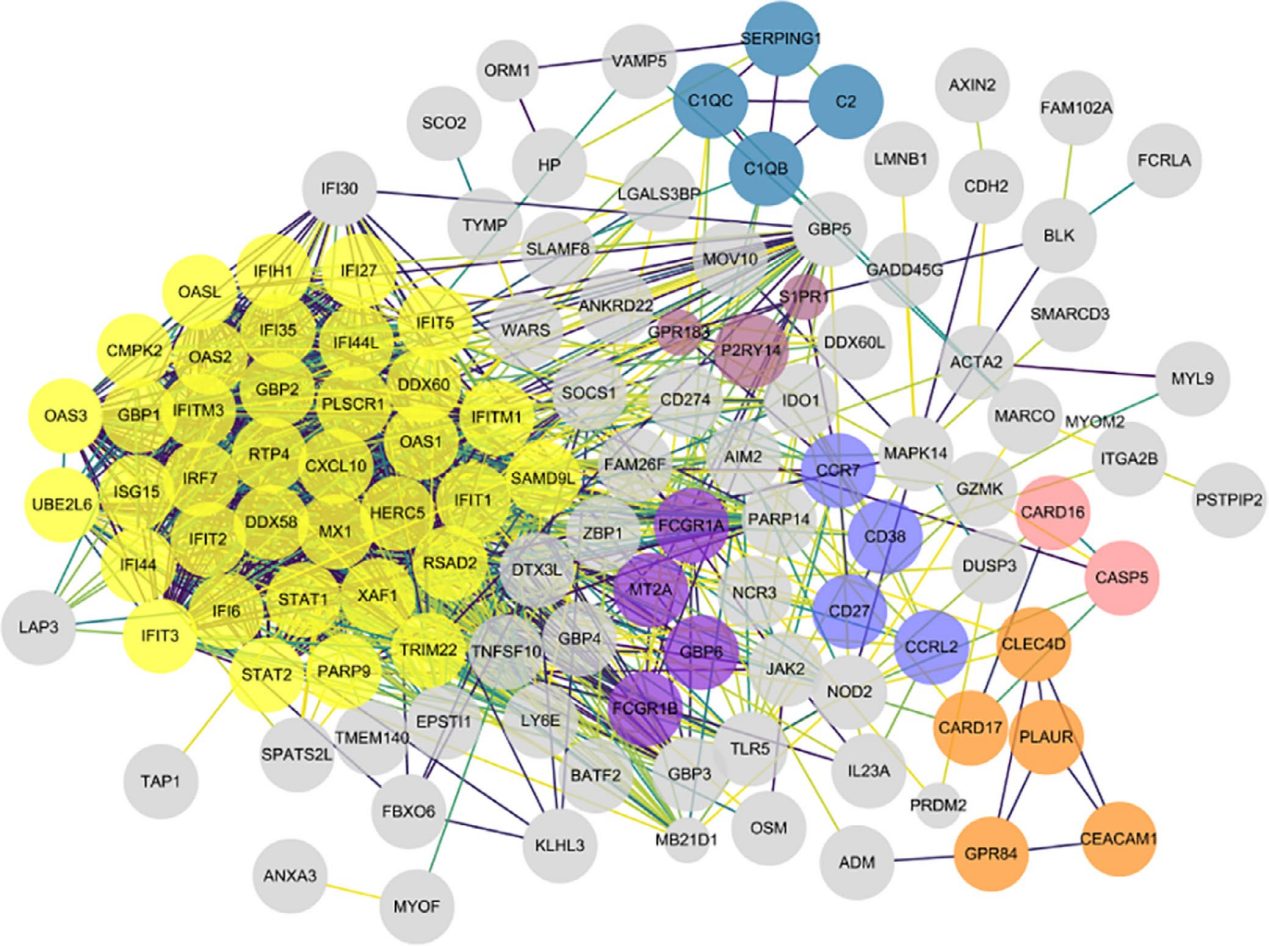
Protein‐protein interaction network was processed with Cytoscape v.3.7.1, and different clusters analysed by MCODE are noted with different colours. The significance of *P*‐value is shown by the size of node. The smaller the *P*‐value is, the larger the diameter of node is. The colour of the edge represents the value of combined score from 0.4 to 1, light to dark

**Figure 8:**
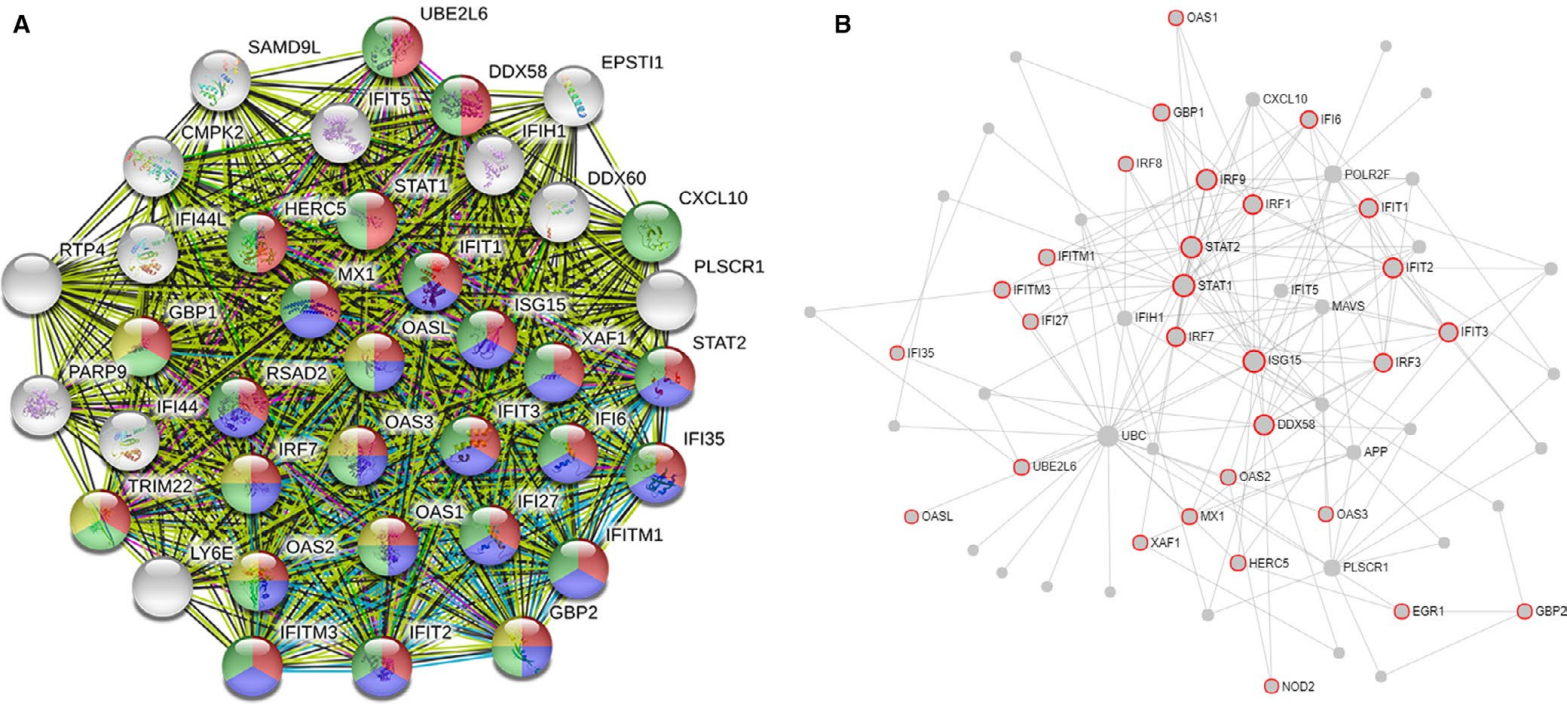
A, STRING analysis shows the interaction between genes in cluster 1, molecules related to interferon signalling are coloured in red, molecules related to interferon‐alpha/beta signalling are coloured in blue, molecules related to cytokine signalling in immune system are coloured in green, and molecules related to interferon‐gamma signalling are coloured in yellow. B, Network analysis was used to validate the results, and molecules involved in cytokine signalling in immune system are noted in red

In order to screen out the core genes from the differentially expressed genes in the PTB sample, 192 differentially expressed genes were uploaded to the STRING for further analysis, and 170 nodes plus 1121 edges were obtained. Local clustering coefficient is 0.503 and PPI enrichment *P*‐value < 1 × 10^−16^, and the data file was then processed with Cytoscape as shown in Figure 7. MCODE was used to process the network data to identify gene clusters (Table 2), genes in the first gene cluster with the highest score were selected for BP enrichment analysis, and it was found that the genes in this gene cluster were mainly involved in defence response and immune system–related function (Table 3). The 38 genes in gene cluster 1 were therefore analysed by STRING and Network Analyst, and results shown in STRING manifested that gene cluster 1 mainly participated in defence response to virus, interferon signalling, interferon‐alpha/beta signalling, cytokine signalling in immune system and immune system–related pathway, all of which were with high statistical significance according to FDR values, and the correlated genes were then marked in STRING as shown in Figure 8A. To further identify which cytokine also plays an important role in the defence of TB infection, we selected genes related to cytokine signalling in immune system in STRING and then verified the result with Network Analyst. Genes related to cytokine signalling in immune system were highlighted as shown in Figure 8B, these genes were intersected with the selected 26 genes in STRING, and a total of 23 genes including GBP2, ISG15 and STAT1 were obtained for further analysis.

**Table 2:** MCODE was used to process the data downloaded from the STRING to further mining gene clusters. Specific data of gene clusters were exported and presented in a tabular form

Cluster	Score (Density*#Nodes)	Nodes	Edges	Node IDs
1	34.973	38	647	RTP4, UBE2L6, IFI44L, PLSCRl, DDX60, IFI6, SAMD9L, OAS2, IFI44, XAFI, IFITM3, PARP9, EPSTil, IFITMl, IRF7, HERC5, GBP2, GBPl, IFIT2, TRIM22, STAT2, OASl, IFI27, RSAD2, IFIT5, IFI35, CMPK2, ISG15, DDX58, STATl, LY6E, IFIHl, IFITl,IFIT3, CXCLlO, OASL, MXl, OAS3
2	4	4	6	FCGRlA, FCGRlB, MT2A, GBP6
3	4	4	6	PLAUR, CLEC4D, GPR84, CEACAMl
4	4	4	6	SERPINGl, C2, CIQB, CIQC
5	3.333	4	5	CCR7, CCRL2, CD27, CD38
6	3	3	3	SlPRl, GPR183, P2RY14
7	3	3	3	CASP5, CARD16, CARD17

**Table 3:** Top 10 biological processes enriched in cluster 1

Biological process	Gene count	False discovery rate
Defence response to virus	29	8.03 × 10^−45^
Response to virus	30	1.44 × 10^−42^
Type I interferon signalling pathway	21	8.30 × 10^−37^
Innate immune response	31	1.67 × 10^−33^
Response to other organism	32	1.40 × 10^−32^
Defence response	35	1.6 × 10^−32^
^Immune response^	32	2.0 × 10^−24^
Cytokine‐mediated signalling pathway	25	5.8 × 10−^24^
Immune system process	35	4.2 × 10^−23^
Response to stress	37	3.00 × 10^−21^

### 3.4 Further miRNA mining and interaction network analysis

**Figure 9:**
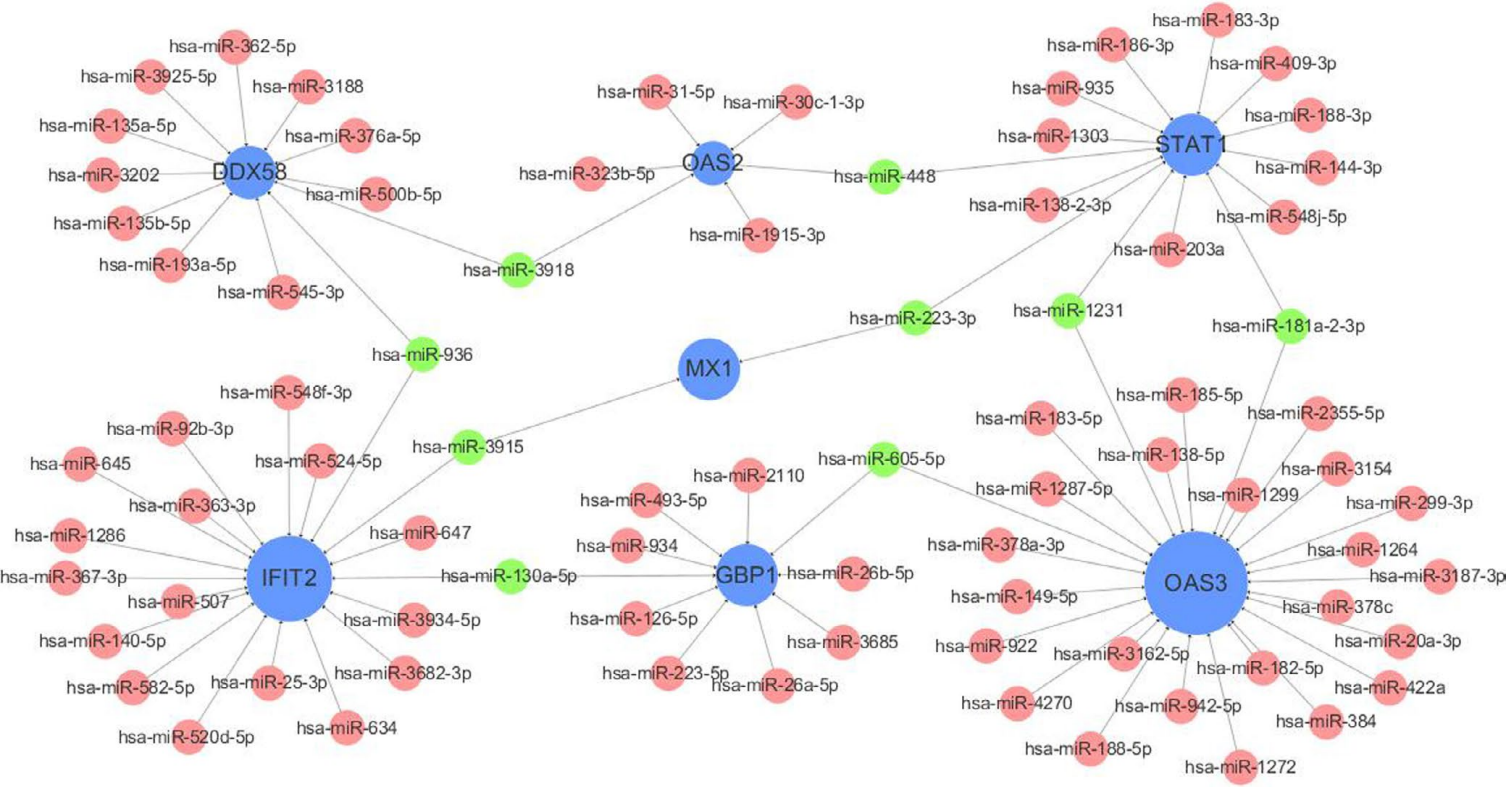
Interaction network between genes involved in cytokine signalling in immune system and its targeted miRNAs. Genes are coloured in blue, and node size is adjusted according to number of targeted miRNAs; miRNAs are coloured in red; miRNAs targeting more than two genes simultaneously are coloured in green

Twenty‐eight genes related to the cytokine signalling in immune system were screened out, and gene‐miRNA analysis was performed with miRWalk 2.0 software. The intersection of miRNA results predicted by TargetScan, miRanda, miRDB, miRWalk and RNA22 databases was selected as the prediction result. The selection conditions were set as *P* < .05, the minimum seed sequence length was 7 mer, and the target gene binding region was 3′UTR. Cytoscape was used to draw the interaction network as shown in Figure 9. miRNAs with high number of gene cross‐links (≥2) were selected (Table 4).

**Table 4:** miRNAs and its target genes

miRNA	Genes targeted By miRNA	Gene count
miR‐130a‐5p	GBP1, IFIT2	2
miR‐605‐5p	GBP1, 0AS3	2
miR‐223‐3p	MX1, STAT1	2
miR‐1231	OAS3, STAT1	2
miR‐3915	IFIT2, MX1	2
miR‐3918	OAS2, DDX58	2
miR‐448	STAT1, 0AS2	2
miR‐936	DDX58, IFIT2	2
miR‐181a‐2‐3p	STATl, OAS3	2

### 3.5 Verification of potential biomarker expression by qRT‐PCR

**Figure 10:**
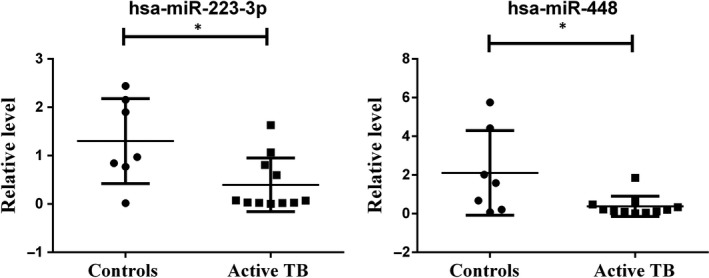
qRT‐PCR results show that the expression levels of miR‐223‐3p (*P*‐value = .016) and miR‐448 (*P*‐value = .021) in plasma of TB patients were obviously lower than that of healthy controls

Nine miRNAs were verified, and it was found that miR‐223‐3p and miR‐448 had high reliability, which all target STAT1. Then, the selected biomarkers including miR‐223‐3p and miR‐448 were validated in TB plasma samples using qRT‐PCR analysis. Consistent with the prediction, the results showed that the expression levels of miR‐223‐3p (*P*‐value = .016) and miR‐448 (*P*‐value = .021) in plasma of TB patients were obviously lower than that of healthy controls (Figure 10).

### 3.6 circRNA prediction

The corresponding circRNA of hsa‐miR‐223‐3p and hsa‐miR‐448 was predicted with StarBase 2.0. The selection threshold was the highest reliability (very high stringency ≥5). After cross‐linking, two circRNAs targeting two key miRNAs were found including SAMD8_hsa_circRNA994 and TWF1_hsa‐circRNA9897.

## 4 DISCUSSION

Tuberculosis is an infectious disease that seriously endangers human health. It is caused by MTB, which parasitizes in macrophages.^19^ In this study, we screened out 192 differentially expressed genes including 156 up‐regulated genes and 36 down‐regulated genes in PTB patients. The genes including BATF2, AIM2, FCGR1B, HP, TLR5 and ANKRD22, and total of 136 genes with significant differences were consistent with the original study.^8^ Then, databases including GSEA, DAVID, Funrich and IPA were used to do gene enrichment analysis, and results show that these genes are mainly involved in interferon signalling pathway and cytokine signalling in immune system. IPA activation *z*‐score also shows that interferon signalling pathway has been highly activated. And STAT1 was found highly related to interferon signalling pathway as well as cytokine signalling in immune system.

Cytokines are small glycoproteins with biological activities secreted by cells, such as interleukin, interferon and tumour necrosis factor, which can act as signalling molecules between cells to mediate the interaction between immune cells and participate in inflammatory reactions.^4^ Cytokines play an important role in cell differentiation, proliferation and immune regulation, and it binds to cell membrane surface receptors to activate intracellular signalling pathways such as the JAK‐STAT signalling pathway and the p53 signalling pathway. Signalling between cytokines and specific cell subsets is key to homeostasis in vivo. In the pathogenesis of TB, interferon signalling is essential for the host immune defence response, and interferon can increase the cell activity of native immune cells including natural killer (NK) cell, cytotoxic lymphocyte (CTL) cell and macrophages. Especially, IFN‐γ secreted by Th1 cells can promote the large secretion of macrophage inflammatory protein‐1α (MIP‐1α) and RANTES, thus chemotactic monocytes clustering around the TB foci to phagocytose, and eliminate MTB. However, MTB can inhibit the macrophage response to IFN‐γ to survive in host cells, so IFN‐γ is important in the defence of MTB infection.^20^, ^21^ In the process of TB infection, IFN I pathway plays a crucial role in anti‐TB infection, but it is also a double‐edged sword, and researches show that IFN I including IFN‐α and IFN‐β can help to promote the TB infection by interfering Th1 immune response and suppressing cytokines such as IL‐1β, TNF‐α and IFN‐γ. However, experiment in mice shows that IFN I can inhibit the activation of macrophage to protect host in the absence of IFN‐γ.^22^ Therefore, balance between IFN I and IFN‐γ is essential in the host immune defence against MTB. As both genes that related IFN I and IFN‐γ are up‐regulated, the detailed interaction between the two pathways is unknown, but has potential research value. Research also shows that interferon (IFN)‐γ release assays (IGRAs) are probably the most accurate tests for the detection of latent TB infection.^23^

In order to validate our results, several bioinformatic analysis tools were used to do the enrichment analyses including gene set and modular enrichment analysis. Interestingly, we found that genes involved in interferon IFN I and IFN II signalling pathway both significantly up‐regulated in current study. GSEA of the down‐regulated genes showed that there was no significant biological indication of the down‐regulated genes, which may be due to too few down‐regulated genes. IPA core analysis also revealed that interferon pathway was significantly activated, and there are 14 genes related to this pathway including STAT1, MX1, OAS1, SOCS1, STAT2, TAP1, IFI6, IFI35, IFIT1, IFIT3, IFITM1, IFITM3, ISG15 and JAK2. Both STRING and Network Analyst show that genes related to IFN I have highest scores, might have inhibited the IFN‐γ. We further analysed genes related to cytokine signalling in immune system to explore core genes and whether it intersects with key genes in the interferon signalling pathway according to IPA.

Meanwhile, we want to know which cytokine pathways also play important roles in the anti‐tuberculosis process besides the interferon pathway; therefore, two databases were combined to screen out the genes with high confidence. Finally, 28 genes were selected to do next step analysis. Reactome pathway analysis shows that cytokine signalling in immune system consists of interferon signalling, signalling by interleukins, growth hormone receptor signalling and TNFR2 non‐canonical NF‐kB pathway. Further miRNA analysis shows that miRNA‐223‐3p and miRNA‐448 with high reliability have a common target gene STAT1, which involved in both interferon‐gamma signalling and IFN I signalling. Meanwhile, IPA core analysis also shows that in the top upstream regulators, STAT1 plays a leading role, and has been highly activated, with a *P*‐value = 9.81E‐61 and *z*‐score = 6.392. In fact, in the early stage of TB infection, STAT1 can promote the activation of transcription by downstream apoptotic factors through phosphorylation. However, with the prolongation of infection time, the intracellular non‐phosphorylated STAT1 protein increased, and these non‐phosphorylated STAT1 proteins can enhance the expression of anti‐apoptotic protein McL‐1 and inhibit the phosphorylated kinase JAK1 of STAT1, and research also shows that it can inhibit the CD95/CD95l‐mediated apoptosis in macrophages and destroy the stability of the pro‐apoptotic complex eEF1A/IFIT1, thus causing the immune escape of MTB.^24^ Meanwhile, STAT1 is also extremely important in promoting macrophages polarization to M1‐polarized macrophages, which can remove MTB through infection more effectively than M2‐polarized macrophages.^25^ STAT1 can bind to specific phosphotyrosine‐containing peptide segments, and when STAT is phosphorylated, it aggregates into homologous dimers to participate in the signal pathway initiated by IFN‐γ. When STAT enters the nucleus, the IFN‐induced early gene expression is activated by the interferon‐gamma activation sequence bound to the promoter.^26^ Therefore, we think that STAT1 may act as a crucial part in the process of TB immune defence procedure. The results of our observation also indicated that cytokine signalling especially the interferon signalling is the most important approach in host defence response of TB infection.

miRNAs are endogenous non‐coding RNA molecule targeting the 3'UTR region of genes with a length of 18‐22 nt and can regulate gene expression at a post‐transcriptional level to degrade or inhibit the translation of target genes.^27^ However, the expression level of miRNAs also infected by its upstream molecular circRNAs, which are non‐coding RNA molecules that do not have a 5' cap end and a 3' tail and are covalently bonded to form a ring structure, and circRNAs act as miRNA sponge, which contains miRNA response elements and can bind to miRNA, preventing miRNA from inhibiting target genes, therefore up‐regulating corresponding gene expression.^28^ And due to its important role in cell information regulation, circRNAs have attracted much attention as new molecular markers in recent years.^29^

In our study, we observed 9 miRNAs that target at least 2 genes which involved in cytokine signalling in immune system. However, only miRNA‐223‐3p and miRNA‐448 can predict the corresponding upstream circRNAs among these selected 9 miRNAs. Low expression of miR‐223‐3p can delay the progression of pulmonary hypertension, whereas high expression of miR‐223‐3p can suppress cell proliferation and migration in lung squamous cell carcinoma via miR‐223‐3p‐mutant p53 regulatory feedback loop.^30^, ^31^ Studies of miR‐223 have shown that it is significantly up‐regulated in macrophages infected with MTB and affects the function of macrophages through negative regulation of NF‐κB activation as well as inhibition of cytokine production.^32^ Furthermore, miR‐223 is responsible for the regulation of matrix metalloproteinases (MMPs), which involved in lung matrix degradation and bacteria dissection via MTB infection. 33miR‐223 was found up‐regulated in active TB patients, and the high‐level expression of miR‐223 can inhibit the apoptosis of macrophages and probably involved in the modulation of innate immune response due to its high enrichment of the induction of pathways in cancer together with miR‐146a, miR‐155, miR‐423‐3p and miR‐21‐5p.^34^, ^35^ Another research shows that expression levels of miR‐223 from both peripheral blood (PBMC) and pleural fluids (PFMC) of patients decreased significantly, therefore may suppress the inflammatory response.^36^ Our results shows that hsa‐miR‐223‐3p has down‐regulated and the inflammatory response pathway has been highly activated according to IPA core analysis and the molecule function analysis shows that in the inflammatory response module, although immune response and antiviral response parts are highly activated, the inflammation part is inhibited in an overall level. However, a study has shown that hsa‐miR‐223 is up‐regulated in patients with tuberculosis (no more than 2 weeks of chemoprophylaxis), while there is no significant change in patients with latent tuberculosis.^37^ It is speculated that the differential expression level of miR‐223 might be related to the differences in the infected TB strains.^38^ The specific molecular mechanism remains to be further studied. Studies on the role of miR‐448 in TB infection are scarce, and some reports show it is relevant to cell proliferation and migration.^39^ In our study, the lower expression of miR‐223‐3p and miR‐448 indicated that a mRNA‐miRNA‐circ_‐_RNA interaction chain may be present in patients with pulmonary TB; therefore, with STAT1 highly expressed, the circRNA would have potential value of further detection.

The potential value of circRNAs as a novel biomarker for disease diagnosis has been confirmed, and some of these circRNAs were found up‐regulated in active TB patients and are related to cytokine‐cytokine receptor interaction and chemokine signalling pathway, and yet some circRNAs including hsa_circRNA_091692, hsa_circRNA_102296, hsa_circRNA_029965 and hsa_circRNA_103571 were reported down‐regulated in active TB patients.^40^, ^41^, ^42^ As our study has confirmed that hsa‐miR‐223‐3p and hsa‐miR‐448 are significantly down‐regulated in patients with pulmonary TB, SAMD8_hsa_circRNA994 and TWF1_hsa_circRNA9897 have high potential value to be used as novel biomarkers. Hence, our results lay a foundation for further researches.

Based on our current study, we found that cytokine signalling in immune system especially interferon signalling pathway is extremely important through TB infectious disease. Therefore, we identified molecules that have significant relevance with interferon signalling, and STAT1 along with its related miRNAs, and circRNAs including miR‐223‐3p, miR‐448, SAMD8_hsa_circRNA994 and TWF1_hsa_circRNA9897 were found as potential biomolecules in the host defence response to TB infection. Further qRT‐PCR results show that hsa‐miR‐223‐3p and hsa‐miR‐448 both down‐regulated in patients with pulmonary TB compared with healthy controls. We believe that through the regulatory networks of these molecules, the interferon signalling pathway of macrophage can be affected.

## CONFLICT OF INTEREST

All the authors declare that there are no conflicts of interest relevant to this article.

## AUTHORS CONTRIBUTIONS

Yu‐rong Fu and Xing‐hao Yi designed the experiments, Zheng‐jun Yi together with Xing‐hao Yi performed the experiments, Xing‐hao Yi wrote the manuscript and analysed the data, and Bo Zhang collected the samples and delivered them.

## Data Availability

Data sets used and analysed during the current study are available from the corresponding author on reasonable request.
